# Normothermic regional perfusion (NRP) use in controlled donation after circulatory determination of death (cDCDD): results of the European Society for Organ Transplantation Bucharest consensus conference

**DOI:** 10.3389/ti.2026.16391

**Published:** 2026-07-22

**Authors:** Eduardo Miñambres, Marius Berman, Marta Velia Antonini, Jose Luis Campo-Cañaveral De La Cruz, Kristopher Croome, Giuseppe Feltrin, Amelia Hessheimer, Carl Jorns, Simon Messer, Anji Wall, Beatriz Domínguez-Gil, Dominique Martin, Gabriel Oniscu, Umberto Cillo

**Affiliations:** 1 Donor Transplant Coordination Unit, Service of Intensive Care, Hospital Universitario Marques de Valdecilla, Santander, Spain; 2 Department of Transplantation, Royal Papworth Hospital NHS Foundation Trust, Cambridge, United Kingdom; 3 Department of Medical and Surgical Sciences (DIMEC), Universita di Bologna, Bologna, Italy; 4 Department of Thoracic Surgery and Lung Transplantation, Hospital Universitario 12 de Octubre, Madrid, Spain; 5 Department of Transplant, Mayo Clinic in Florida, Jacksonville, FL, United States; 6 National Transplant Center, Istituto Superiore di Sanita, Rome, Italy; 7 General and Digestive Surgery Service, Hospital Universitario La Paz, Madrid, Spain; 8 Transplantation Surgery, Karolinska Universitetssjukhuset, Stockholm, Sweden; 9 Division of Abdominal Transplantation, Baylor Simmons Transplant Institute, Dallas, TX, United States; 10 Organización Nacional de Trasplantes, Spanish Ministry of Health, Madrid, Spain; 11 School of Medicine, Deakin University, Geelong, VA, Australia; 12 Division of Transplantation Surgery, Karolinska Institutet, Stockholm, Sweden; 13 Department of Surgery, Oncology and Gastroenterology (DISCOG), University of Padua, Padua, Italy; 14 Hepato-Bilio-Pancreatic Surgery and Liver Transplantation Unit, Azienda Ospedale Universita Padova, Padua, Italy

**Keywords:** consensus conference, DCDD (donation after circulatory determination of death), ESOT, liver transplant, NRP

## Abstract

Normothermic Regional Perfusion (NRP) is emerging as a game-changer in enhancing outcomes for Donation after Circulatory Determination of Death (DCDD). NRP maintains physiological conditions through perfusion with oxygenated blood, outperforming conventional super-rapid recovery techniques significantly improving outcomes and organ utilization. Despite its clinical benefits, widespread adoption of NRP is impeded by heterogeneous organizational, legal, and ethical frameworks. At the ESOT Bucharest Consensus Conference, leading experts in transplantation achieved consensus on 130 relevant NRP-related open issues to facilitate its implementation and guide global practice. Key recommendations include criteria for adoption of NRP, minimal requirements, procedures to be adopted before and during NRP, donor organ evaluation criteria and sequence of organ harvesting. Consensus extends to procedural components (including the configuration of perfusion parameters and strategic team coordination), ethical integrity of NRP in the context of the dead donor rule and key unmet needs for future developments. While significant strides were made in unifying practice, unresolved issues regarding maximum warm ischemic time and variability in legal standards indicate avenues for future research. This consensus underscores the imperative for global standardization in NRP application, promising to elevate the success rates of organ transplants and establish NRP as a foundational element in the evolution of DCDD.

## Introduction

Normothermic regional perfusion (NRP) has revolutionized organ utilization and graft outcomes following donation after circulatory determination of death (DCDD). NRP involves the *in situ* regional perfusion of the abdomen or chest and abdomen with oxygenated blood at normothermic temperature range (35 °C–37 °C), initiated after the formal declaration of death and prior to organ recovery [1].

The growing adoption of NRP in DCDD is primarily due to significant improvements in post-transplant outcomes and organ utilization compared to the conventional super rapid recovery (SRR) technique [2–8]. Unlike organ-specific *ex situ* perfusion technologies, NRP allows for the simultaneous perfusion of multiple organs, thereby potentially reducing costs, complexity and improving patient access to high-quality DCDD organs [5, 9, 10].

Broader implementation of NRP remains limited by organizational, legal, and ethical challenges. Most notably, the absence of standardized criteria and definitions for many technical and clinical aspects are key barriers to its global expansion. This heterogeneity hinders the comparison of outcomes and the harmonization of protocols across centers and countries.

A panel of international experts with extensive experience in the field of organ transplantation and particularly in the use of NRP participated in this Delphi study (cfr. Table 1 and Martin et al.; [11]) specifically focused upon controlled DCDD.

**TABLE 1:** Demographics of panelists.

Characteristic	All panellists (n = 31)
Personal characteristics	
Age	
18–30 years	0%
31–40 years	22.6%
41–50 years	41.9%
51–60 years	29.0%
>60 years	6.5%
Gender	
Male	64.5%
Female	35.5%
Country of practice	
Belgium	3.2%
France	9.7%
Italy	16.1%
Netherlands	6.5%
Norway	3.2%
Spain	16.1%
Sweden	9.7%
United Kingdom	12.9%
United States	22.6%
Professional profile	
Primary specialty	
Anaesthesiologist	3.2%
Ethicist	3.2%
Intensivist	9.7%
Transplant surgeon	71.0%
Transplant or donation coordinator	9.7%
Other^1^	12.9%
Primary area of practice	
Adult medicine	80.6%
Both paediatric and adult medicine	19.4%
Experience	
Experience with deceased donation or transplantation	
≥10 years	74.2%
5–9 years	22.6%
<5 years	3.2%
Experience with normothermic regional perfusion (NRP)	
≥10 years	32.3%

Values are percentages of all panellists who participated in survey waves 1 and 2 (n = 31). Percentages may not sum to 100% owing to rounding.

1 Includes respondents whose primary specialty did not fall into the listed categories and, namely,: Cardiovascular Perfusionists, Nephrologists, Transplant Hepatologist.

The objective was to identify and analyze areas of ongoing controversy regarding the application of NRP where there was a lack of standardized consensus across centers and countries (see Results for participant details). Therefore, the aim of this consensus was to address this lack of uniformity by proposing consensus-based recommendations to facilitate the safe and effective global implementation of NRP in controlled DCDD.

## Methods

The Delphi process applied in the overarching DCDD Consensus project has been outlined in detail [11]. In essence, a steering committee of 10 members with expertise in NRP led by two coordinators, developed a questionnaire addressing a range of topics identified as priorities through literature review and group discussions. The committee also identified expert panelists according to the previously described criteria [11], who were invited to participate in the Delphi process. The two coordinators did not participate in the expert panel.

Two survey waves were conducted using an online questionnaire administered by the independent company Adelphi Targis. All panelists who completed the first questionnaire were invited to complete the second wave survey. The questionnaire was refined for the second wave as described [11].

In each survey round panelists indicated their level of agreement with a series of statements using a Likert scale (1–9, strongly disagree–strongly agree); responses were analysed using descriptive statistics with ‘disagreement’ assigned to ratings 1–3, “neither agree nor disagree” 4-6, and “agreement” to ratings 7–9. Participants could indicate if a question was not relevant to their expertise and their responses were removed from the denominator when evaluating consensus on specific questions. Statements that reached 75% agreement were deemed to achieve consensus.

Consistent with the design of the Bucharest consensus, paediatric DCDD was addressed separately by a dedicated panel.

## Results

Thirty-one experts from nine countries completed the first round of the Delphi process, all of whom completed the second round. Panel demographics are shown in Table 1.

Consensus was achieved on 105 statements after the first round, and on an additional 25 statements after the second round. Following the first and the second round, 52 and 74 statements respectively were eliminated due to lack of consensus. In the second round 48 statements of the first round were reproposed or remodulated whereas 25 new statements were added. The recommendations summarised in Tables 2–14 below reflect the statements for which consensus was achieved, noting some were combined for efficiency.

**TABLE 2:** Consensus recommendations regarding some DEFINITIONS in the specific context of NRP adoption.

a. The best term used to describe donation that occurs following the withdrawal of life sustaining measures (WLSM) and circulatory arrest is “donation after circulatory determination of death” (DCDD)
b. Circulatory arrest refers to the absence of pulsatile blood flow following WLSM.
c. Acirculatory phase refers to the period between circulatory arrest and the start of cold preservation OR, when used, normothermic regional perfusion
d. Total warm ischaemia time begins when life sustaining measures are withdrawn and ends with the start of cold preservation, or, when used, normothermic regional perfusion
e. Functional warm ischaemia time refers to the period between the onset of sustained organ hypoperfusion (as specified by local or national guidelines) and the start of cold preservation, or, when used, normothermic regional perfusion
f. The no-touch period refers to the mandated minimum period of time following circulatory arrest before death is declared and organ retrieval may begin
g. Functional perfusion of an organ refers to the passage of blood or other perfusates through blood vessels that results in exchange of oxygen, solutes, and/or nutrients to the cells of the organ and removal of cellular waste

**TABLE 3:** Consensus recommendations regarding the ADOPTION of Machine Perfusion Technologies.

1. Machine perfusion should be used in order to improve early and/or late postoperative graft function. Machine perfusion (*in situ* NRP, *ex situ* NMP, and/or *ex situ* hypothermic machine perfusion (HMP) should be routinely used for all cDCDD donors
2. To improve postoperative graft function, NRP is recommended in the cDCDD context: a) In case of expected long warm ischaemic times, e.g., in countries with a no-touch time >5 min b) In donors with a high donor risk index.12 c) When organs may need more comprehensive evaluation prior to transplantation d) In donors with end stage cardiac failure undergoing extracorporeal membrane oxygenation (ECMO) e) In all cDCDD cases following withdrawal of life-sustaining measures (WLSM) f) When donation occurs following euthanasia

**TABLE 4:** Consensus recommendations regarding the A-NRP (Abdominal Normothermic Regional Perfusion) and TA-NRP (Toraco-Abdominal Normothermic Regional Perfusion) TEAM COMPOSITION and TRAINING.

1. The A-NRP and TA-NRP team should include at least the following: a) 1 healthcare professional fully competent in performing NRP cannulation and 1 assistant b) 1 perfusionist or specialist fully competent in operating an NRP circuit c) 1 scrub nurse d) 1 circulating nurse e) 1 transplant coordinator/donor coordinator
2. To minimise technical adverse events and to improve postoperative graft outcomes a) New NRP teams should be supervised by a tutor until the team members have been determined to be competent b) NRP team members should have completed formal NRP-specific training under NRP experts’ supervision c) The retrieval surgeons should complete NRP-specific training

**TABLE 5:** Consensus recommendations regarding the MINIMAL EQUIPMENT REQUIRED in the routine cases to complete NRP safely.

a. A centrifugal pump
b. For A-NRP, a membrane lung with integrated heater exchanger
c. For TA-NRP, a membrane lung with integrated heater exchanger
d. A heater unit
e. A flowmeter
f. A bubble sensor
g. A pressure sensor

**TABLE 6:** Consensus recommendations regarding PROCEDURES PRIOR and THOUGHOUT NRP.

1. Ante mortem procedures prior to NRP a) Donor heparinisation is recommended to improve NRP success (if permitted by local law and guidelines) b) Antemortem insertion of guidewires, is recommended if permitted by local law and guidelines) c) For TA-NRP, femoral cannulation should be performed (if permitted by local law and guidelines)
2. Before starting NRP, to reduce complications and improve graft outcomes a) A team briefing and handover should be performed, ensuring that all members understand their responsibilities b) Volume replacement (blood or colloid) should be readily available to correct any blood/volume loss c) The circuit should be primed to prevent subsequent air embolization
3. NRP priming solution or NRP perfusate after NRP initiation should include the following components in routine TA-NRP and A-NRP cases: a) Balanced crystalloid solution b) Heparin
4. Throughout NRP, to minimise technical adverse events and improve postoperative graft outcome a) The heater temperature should be 35 °C–37 °C b) Blood gas analysis should be performed 3–4 min after NRP is functioning c) The oxygen/air mixture should be titrated according to blood gas results d) The starting oxygen/air mixer should deliver gas flow at around 2 or 3 L per minute e) The oxygen/air mixer should be checked regularly after NRP is started f) A flowmeter must be used during NRP to guarantee an optimal Extracorporeal blood flow (EBF) g) Effective communication between team members should be ensured h) Meticulous haemostasis, particularly in the chest, to prevent blood loss and circuit failure should be guaranteed i) The risk of venous or arterial clots that may impair/prevent adequate venous return should be minimized j) Hyperoxemia, which promotes the formation of reactive oxygen species thus exacerbating organ reperfusion injury, should be avoided k) Serial blood gases at NRP initiation and at least every 30 min throughout NRP should be measured I) The functioning of the membrane lung should be verified at least once. m) Any sudden volume loss, which can cause sudden loss of venous return and NRP failure (if a reservoir is being used) should be timely identified and corrected
5. After NRP has been initiated, it is recommended to use plasma substitutes or packed red blood cells if more volume is required during NRP

**TABLE 7:** Consensus recommendations to improve the TECHNICAL SUCCESS of A-NRP and post-operative outcomes.

a) …The thoracic descending aorta is clamped (with a surgical clamp or intra-aortic balloon) before starting A-NRP and throughout the procedure
b) …The thoracic descending aorta should not be clamped and cut with active drainage before starting A-NRP and throughout the procedure
c) …It must be ensured that the coeliac axis is not blocked by the clamping

**TABLE 8:** Consensus recommendations on requirements for TA NRP and to IMPROVE the TECHNICAL SUCCESS of TA-NRP and post-operative outcomes.

1. Requirements a) …To use a hybrid (open) ECMO circuit with a rigid reservoir b) …That all potential TA-NRP donors will have a dedicated computerized tomography scan (CT) pre-treatment withdrawal c) …That if no CT is available, the retrieval team needs to dissect the aortic arch distal to the left subclavian to ensure no aberrant right subclavian artery
2. To improve the technical success it is recommended that a) …If prolonged time is expected to be required for sternotomy and arch vessels clamping, consider initiating A-NRP in the meantime, providing peripheral cannulation and thoracic descending aorta balloon (particularly if antemortem interventions are allowed before WLSM) b) …The aortic arch vessels are clamped or ligated c) …If A-NRP is initiated before sternotomy and aortic arch vessel clamping, and an endovascular balloon is used, the clamp must be placed below the left subclavian artery, e.g., by using a left radial arterial catheter

**TABLE 9:** Consensus recommendations to PRESERVE post-operative lung/heart GRAFT FUNCTION following TA-NRP.

a) Excessive transfusion of packed red cells is avoided
b) Excessive fluid administration is avoided
c) Cardiac distension is prevented
d) The risk of aortic dissection during cannulation of the aorta is reduced by ensuring the placement of the tip well into the aortic arch
e) A centrally inserted dual-stage venous cannula is used, which provides superior venous compression as compared with femoral cannulation, thus mitigating the risks of failed cardiac resuscitation and lung congestion
f) TA-NRP is withdrawn as soon as possible if cardiac function is optimal, to allow recovery of the “stunned heart”

**TABLE 10:** Consensus recommendations regarding the EVALUATION of DONOR ORGANS in donors undergoing NRP.

1. During A-NRP a) In both TA-NRP and A-NRP, the contents of the thoracic cavity should be thoroughly inspected for neoplasms (especially of the lung and oesophagus) and other pathologies b) Macroscopic evaluation of the organs should be performed c) If the liver appears congested and feels stiff at the start of A-NRP, this does not indicate that the liver is non-viable d) During NRP, normal colour should return to the liver, and stiffness should decrease e) Macroscopic evaluation of organ viability should be supplemented with laboratory evaluation f) When interpreting laboratory parameters, it is important to note that administering a large volume of fluids can dilute serum markers, making them appear misleadingly lower g) Serial transaminases and serial lactate are useful ancillary tests to assess liver injury h) There is no useful biochemical marker for kidney function during NRP i) Complete cessation of urine output does not indicate that the kidneys are non-viable j) If pancreatitis is suspected on macroscopic examination of the pancreas, serum amylase is a useful ancillary test
2. During TA-NRP a) Bronchoscopy is a useful ancillary test for lung viability b) *Ex Vivo* lung perfusion (EVLP) should be used for lung evaluation or reconditioning following the general criteria for this technique c) Cardiac function can be reliably assessed by pulmonary arterial catheterisation with serial cardiac output measurements d) Cardiac function can be reliably assessed with esophageal echocardiography e) Cardiac function can be reliably assessed by visual assessment with brief volume loading without circulatory support, looking for right ventricular dysfunction, bradycardia, malignant arrhythmia and rises in blood pressure

**TABLE 11:** Consensus recommendations about the SEQUENCE of donor organ recovery.

1. a) If thoracic and abdominal organs are to be recovered simultaneously, detailed pre-operative planning is required to delineate roles and responsibilities of each team, including the timing and sequence of organ recovery b) The heart should be recovered first, followed by the liver, lungs and remaining abdominal organs c) The liver should be recovered prior to the pancreas and kidneys d) If thoracic and abdominal organs are to be recovered simultaneously, two separate surgical teams are required (one for thoracic organs and one for abdominal organs) 2. Recovery of the heart should be abandoned, and attention refocused on recovery of lungs and abdominal organs, if TA-NRP cannot be stopped after an agreed (zonal or national protocol) perfusion time has elapsed and there is metabolic and/or macroscopic evidence that cardiac function cannot sustain systemic perfusion

**TABLE 12:** Statements about ethical considerations of NRP with regards to the dead donor rule.

a) NRP should not be used if it violates the “dead donor rule” (DDR). The DDR is the ethical requirement that donors must be lawfully determined to be dead prior to removal of vital organs
b) Appropriate techniques must be used to effectively and completely block prevent all blood flow to reperfusion of the brain during NRP to ensure that the permanency standard required for the determination of death is maintained
c) Global standards addressing the ethical and legal implications of TA-NRP should be established
d) AGAINST regardless of how the law defines death, thoracic NRP should not be used because the act of restoring circulation to the heart violates the DDR
e) AGAINST regardless of how the law defines death, thoracic NRP should not be used because re-initiation of cardiac activity violates the DDR
f) AGAINST regardless of how the law defines death, thoracic NRP should not be used because it is currently impossible to exclude the possibility of cerebral (re)perfusion, and thus it violates the DDR
g) AGAINST regardless of how the law defines death, thoracic NRP should not be used because the public may perceive the restoration of circulation in the body as a breach of the intent of the DDR, even if it is not technically a breach of the rule
h) AGAINST regardless of how the law defines death, A-NRP should not be used because the act of restoring circulation to part of the body violates the DDR
i) AGAINST regardless of how the law defines death, A-NRP should not be used because it is currently impossible to exclude the possibility of cerebral (re)perfusion, and thus it violates the DDR
j) AGAINST regardless of how the law defines death, A-NRP should not be used because the public may perceive the restoration of circulation in the body as a breach of the intent of the DDR, even if it is not technically a breach of the rule

**TABLE 13:** Consensus recommendation on how to ENSURE the BRAIN is NOT PERFUSED during TA-NRP.

1. a) Appropriate techniques must be used to confine oxygenated perfusion to organs intended for transplantation and maintain cessation of oxygenated perfusion of the brain, completely and successfully. b) Effective techniques must be used to maintain cessation of all cerebral perfusion during NRP c) Effective techniques must be used to maintain cessation of oxygenated perfusate to the brain during NRP d) During TA-NRP, clamping or ligation of the aortic arch vessels that perfuse the brain and sectioning of the cephalic ends of the vessels* plus drainage and aspiration of blood from their caudal ends to achieve atmospheric pressure in the arteries is sufficient to maintain cessation of cerebral perfusion * See discussion section
2. To maintain cessation of cerebral perfusion during TA-NRP in settings where the no touch period is less than or equal to 5 min, at a MINIMUM a named person in the team should be responsible for determining that TA-NRP has been unsuccessful and shifting the team’s focus to salvaging lungs and abdominal organs
3. A written contingency plan should be included in all TA-NRP protocols specifying the procedural steps to follow to rapidly abort TA-NRP, in case blood flow to the brain or any signs of cerebral activity are detected

**TABLE 14:** UNMET NEEDS and FUTURE RESEARCH (reaching consensus).

a) There is a lack of standardisation regarding TA-NRP practices between countries
b) There is a lack of standardisation regarding A-NRP practices between countries
c) There is a lack of standardisation regarding NRP use between countries because of different laws between countries
d) More research is needed to determine the optimal organ preservation solution
e) More research is needed to inform an international consensus on the acceptable cut-off values for laboratory parameters to assess donor organ function, especially for liver markers
f) More research is needed to inform an international consensus on the most reliable methods to evaluate the viability of a donor heart in the context of NRP
g) In future research studies regarding NRP management techniques, the following metrics should be incorporated to assess effectiveness
- Organ utilisation rate
- Graft survival rate
- Recipient survival rate

### Definitions in NRP adoption

The most appropriate term to describe donation following the withdrawal of life sustaining measures (WLSM) and subsequent circulatory arrest is donation after circulatory determination of death (DCDD) [12]. In this consensus, a high level of agreement was achieved on most of the definitions (Table 2). Terminology is discussed in a greater detail in the overview article on consensus methodology [11].

Although the group agreed on the definition of “functional warm ischemic time”, no consensus was reached regarding the specific hypoperfusion thresholds that delineate the onset of this period, and this is a remarkable point to discuss in the future to allow comparisons of results between countries and centers.

### Adoption of machine perfusion

Provided that the panelists strongly agreed on the need of a routine use of Machine Perfusion (MP) to improve results in cDCDD, it should be remarked that sophisticated preservation techniques are not absolutely mandatory for a DCDD program to exist and provide appropriate outcomes even though NRP has shown, with intermediate level of evidence, to provide better recipient outcomes [8].

### Team composition and training

There is strong consensus that a NRP team should include at least 5 different fully competent professionals (Table 4). This integrates what recommended in a recent ESOT consensus document on the topic [13]. The expansion of NRP programs requires well-prepared and adequately trained teams. There is clear agreement that new teams must achieve competence through a supervised training phase under expert oversight [14].

However, no consensus was reached regarding the minimum number of procedures required to achieve competent status within scientific societies or health authority organized training programs. These considerations should be taken into account when designing educational activities on the application of NRP in cDCDD.

There was also broad consensus on the minimum equipment required to perform NRP safely in routine clinical settings (Table 5).

### NRP procedures

The legal framework governing premortem interventions varies considerably across countries [15, 16]. Nevertheless, there is broad consensus that the administration of heparin to the donor and the antemortem insertion of guidewires or cannulation, prior to initiating NRP, enhances the likelihood of optimal graft function. Indeed, antemortem insertion of guidewires or cannulation may help reducing the duration of warm ischemic time. However, such practices should only be performed if permitted under local laws and institutional guidelines. Interestingly, in the case of TA-NRP, but not in the A-NRP, peripheral cannulation before WLSM was generally considered appropriate (Table 6).

A team briefing and handover is recommended to ensure that all members understand their responsibilities. Volume replacement should be administered in cases of blood or fluid loss, to prevent NRP failure, always ensuring adequate hemoglobin levels (>8 mg/dL) to avoid inadequate oxygen delivery [17]. There is strong consensus on the recommended target parameters (Table 6, point 4). Adequate anticoagulation during NRP is essential for optimal procedural performance. However, no consensus was reached on the minimum monitoring frequency.

During multiorgan retrieval with NRP, chest bleeding after lung retrieval is a major concern in combined thoracic and abdominal recovery [18, 19]. Meticulous haemostasis, particularly in the chest, is highly recommended to prevent blood loss and circuit failure.

The NRP priming solution or perfusate after initiation should include a heparin-balanced crystalloid solution. No other components have been recommended (Table 6, point 3). If additional volume is required during NRP, plasma substitutes or packed red blood cells should be used.

### Technical success of NRP

The optimal duration of NRP remains unclear in the literature. NRP is typically maintained for 1–2 h. Some retrospective studies have suggested no significant differences in outcomes between 1 and 4 h of perfusion [20–22], but, differently from previous consensuses [17], no consensus on the optimal duration was reached by the expert group. It was unequivocally agreed that abdominal NRP should never be initiated without first clamping the thoracic descending aorta, either surgically or via the placement of an intra-aortic balloon [5, 9, 23]. However, there was clear consensus against dividing the thoracic descending aorta with active drainage before starting A-NRP.

The use of TA-NRP for heart recovery in DCDD donors has expanded worldwide. To maximize the success of all retrieved organs, if prolonged time is anticipated for sternotomy and aortic arch vessel clamping, it is recommended to initiate A-NRP in the interim, using peripheral cannulation and a thoracic descending aortic balloon (particularly when antemortem interventions are permitted), as previously described [24, 25].

A major barrier to the global adoption of TA-NRP is the risk of restoring cerebral circulation following the declaration of death. It has been agreed that the aortic arch vessels must be clamped or ligated before starting TA-NRP (Table 8, point 2). However, no consensus was achieved on whether the vessels should also be transected and left to drain to atmospheric or negative pressure. This remains a point of contention, underscoring the need for further studies to clarify whether transection or drainage to negative pressure of the supra-aortic trunks is essential to definitively remove any risk of postmortem cerebral reperfusion. After the consensus emerged that in the United States for both A-NRP and TA-NRP venting above the clamp is now mandatory [26] but this is not the case for many other realities. Consensus was reached on several refinements regarding the TA-NRP technique (Table 8, point 1).

### Preservation of lung/heart function

The combined retrieval of lungs and heart using TA-NRP presents significant technical challenges. To preserve optimal post-transplant lung and cardiac function, it is recommended to avoid excessive fluid transfusion and prevent cardiac distension during the procedure. If A-NRP is initiated before sternotomy and arch vessel clamping and an endovascular balloon is used, the balloon should be positioned below the left subclavian artery. Once optimal cardiac function is achieved, TA-NRP should be discontinued as soon as possible to facilitate the recovery of the stunned heart (Table 9).

### Evaluation of donor organs in NRP

It is widely acknowledged that macroscopic assessment of the graft by the retrieval surgeon is indispensable for organ validation and may be supplemented, when appropriate, by complementary laboratory investigations. However, the routine histopathological (microscopic) evaluation of grafts and routine biopsies for the evaluation of kidneys and liver were not considered necessary.

There serial transaminases and serial lactate were agreed to be useful ancillary test to assess liver function during NRP. Traditionally, a marked elevation of serum aminotransferase (AST, ALT)—typically a threefold or greater increase from baseline levels (less decisive for many nowadays) — and a progressive rise in lactate have been interpreted as suggestive of an ischemic injury and suboptimal perfusion with high risk of primary non-function (PNF) [21, 27–29].

For pulmonary assessment, bronchoscopy was agreed as a valuable adjunctive tool for determining graft suitability. EVLP is an option for donor lungs that cannot be properly evaluated in the donor and/or for logistical reasons [30]. The consensus considered that EVLP should be recommended for lung evaluation or reconditioning only in the special cases, and not for routine cases (Table 10, point 2).

For heart retrieval using TA-NRP, the assessment of the heart has usually been performed with pulmonary arterial catheterization with serial cardiac output measurements and/or also with esophageal echocardiography [25, 31]. All consensus is detailed in Table 10, points 1, 2.

### Sequence of organ recovery

The simultaneous retrieval of thoracic grafts (lungs and/or heart) and abdominal grafts using NRP remains challenging and increases the overall complexity of the procurement procedure, with a heightened risk of graft injury during retrieval [10, 18, 19, 25].

A major challenge in TA-NRP is managing situations where the procedure involves multiorgan recovery which could risk the other grafts because of problems with the heart. Current literature does not clearly define the point at which the heart should be deemed unsuitable for transplantation, and the focus shift toward the retrieval of lungs and abdominal organs. The panelists considered that if TA-NRP cannot be discontinued after an agreed perfusion time, and there is metabolic and/or macroscopic evidence that cardiac function cannot sustain systemic perfusion, the heart should be considered non-viable for transplantation (Table 11, point 2). At that point, the thoracic aorta should be clamped, and the NRP flow reduced to 2.0–2.4 L/min to ensure adequate perfusion of abdominal grafts [9, 25].

The panelists agreed that a designated person in the team should ideally be responsible for determining that TA-NRP has been unsuccessful and shifting the team’s focus to salvaging lungs and abdominal organs.

The agreed liver-pancreas sequence is clearly not the case when retrieval surgeons prefer to adopt the *en bloc* recovery.

Given the limited panel experience on this specific issue, the discussion did not face the appropriate retrieval sequence in cases of intestinal procurement, particularly regarding *en-bloc* harvesting of a multivisceral graft or the anticipation of intestinal retrieval prior to the liver.

One of the most debated aspects in the assessment of DCDD organs is determining the maximum acceptable limit of warm ischemic time (WIT). These limits have been progressively extended as clinical experience has accumulated since the earliest cases [32, 33]. However, a universally accepted cutoff is yet to be establish among experts.

### Ethical issues

The authors slightly rephrased the statement 12b from the original voted form:

“…effectively and completely block all blood flow to the brain during NRP …” Rephrasing was considered opportune for semantic reasons since the verb “block” implies different intent.

Ethical concerns regarding the use of NRP in DCDD [34] include premortem interventions, and the theoretical possibility of restoring brain perfusion after death is declared [11]. This last concern is one of the most important obstacles that have prevented the expansion of NRP [35–38]. There was notably strong consensus among the panelists who disagreed with a series of normative and empirical claims that are often presented as justification for prohibiting NRP (Table 12).

The confidence demonstrated in the ethical integrity of NRP practices with regards to respect for the “dead donor rule”, when relevant clinical safeguards are effectively implemented likely reflects the inherent bias of the panel, who were selected for their experience with NRP.

### Brain perfusion

There was a consensus that effective techniques must be used to maintain permanent cessation of cerebral perfusion and the delivery of oxygenated perfusate to the brain during NRP. Indeed, clamping or ligation of the aortic arch vessels and sectioning of the cephalic ends of the vessels, plus drainage and aspiration of blood to atmospheric pressure during TA-NRP were considered sufficient to ensure this. However, the exclusive ligation of the arch vessels was not considered an effective technique to maintain permanent cessation of brain circulation during TA-NRP (Table 13).

Thought the implementation of a system of brain monitoring during TA-NRP has been suggested to detect any potential failure in maintaining permanent absence of blood flow to the brain, which should immediately prompt the halting of NRP, mainly in those cases where cessation of brain circulation is maintained by only clamping the arch vessels [9, 39], it has not been considered mandatory to use transcranial Doppler, transcranial evoked potentials or invasive arterial blood pressure monitoring of the circle of Willis from WLSM until the end of TA-NRP. In fact, the use of transcranial Doppler during TA-NRP is highly controversial, and it has not been considered the most reliable monitoring technique to ensure no blood flow during TA-NRP.

### Unmet needs and future research

There is a lack of standardization regarding A-NRP and TA-NRP practices between countries, which is concerning. This lack of standardization is considered to be the result of different legal frameworks between countries or the absence of national protocols. In future studies regarding NRP management techniques, organ utilization metrics should be incorporated.

## Discussion

There is broad expert agreement that perfusion techniques—either *in situ* (NRP) or *ex situ* (NMP or HMP)—should be routinely implemented in all cDCDD donors to optimize post-transplant graft function if they are available. In countries unable to incorporate these techniques, SRR and static cold storage can be adopted provided a strict control for other factors that affect post-transplant outcomes.

Recent studies have consistently demonstrated the positive impact of these perfusion strategies on recipient outcomes when compared with the standard rapid recovery technique [3–5, 7, 8, 25]. This Consensus did not aim at any point to assess the combined use of NRP with *ex situ* preservation techniques such as NMP or HMP. This topic has been recently described in the European Liver and Intestine Transplant Association (ELITA) consensus [17].

NRP is strongly recommended in many different situations (Table 3). However, no consensus was reached regarding scenarios of deceased donation involving conscious patients who can make their own decisions about end-of-life care (e.g., persons with end-stage respiratory failure or neurodegenerative diseases, or those in whom medical assistance in dying has been authorized). In countries where DCDD is possible under these circumstances, patients may require intensive care to facilitate organ donation (ICOD), including elective ventilation [40–42]. The limited clinical experience with these specific subgroups of donors and ICOD is likely the main reason for the lack of agreement.

These discrepancies regarding the patient groups in which NRP should be applied highlight the heterogeneity of current practices and underscore the urgent need for consensus statements and evidence-based clinical guidelines. This Delphi Consensus highlights the substantial differences observed in the implementation of NRP worldwide, all the more striking given that the participating experts have extensive experience in this field.

This study demonstrates a high degree of uniformity in the technical and human resource requirements to implement NRP in any center. It also shows broad consensus regarding the training needs of newly established NRP teams.

We believe there is an urgent need to agree on certain terms, such as functional warm ischemic time (FWIT), in order to promote uniformity across studies. Unfortunately, no consensus was achieved in defining the maximum acceptable limit of warm ischemic time (WIT) among experts.

One of the most important aspects of this Consensus is the agreement that the use of certain premortem measures, such as the administration of heparin or the placement of guidewires or cannulas before WLSM, improve post-transplant outcomes. This consensus may help to change the regulatory framework in many countries where these practices are still not permitted [15]. One of the most surprising findings in this area was that peripheral cannulation before WLSM was generally considered appropriate in the setting of TA-NRP, but not in A-NRP (Table 6).

Undoubtedly, the area that generates the greatest disagreement among experts is the use of TA-NRP, where there is considerable divergence regarding how to ensure the absence of cerebral perfusion during the procedure.

Due to the uncertainty that complete cessation of blood flow to the brain results by only clamping the arch vessels, current protocols in the United Kingdom and Spain have added the step of severing the arch vessels distal to the clamps and draining blood from the cephalad ends to atmospheric pressure [39, 43, 44]. However, no consensus was achieved regarding whether the vessels should be transected and left to drain to atmospheric pressure. Some authors, particularly in Europe, argue that clamping alone may be insufficient to ensure the absence of postmortem cerebral perfusion [43–46], whereas U.S. guidelines consider both clamping alone and clamping with transection as acceptable options [45, 47]. The American Society of Transplant Surgeons produced an updated statement supporting clamping and venting the supra-aortic trunks when using TA-NRP [26].

The differences observed between authors, and particularly between European and American guidelines regarding clamping alone or clamping and sectioning the supra-aortic trunks when using TA-NRP represent a point of controversy that will need to be solved [43–47]. A Spanish study evaluating the intracerebral blood pressure at the Circle of Willis and the basilar artery—which encompasses the anterior, middle and posterior cerebral circulations—provides strong evidence to alleviate concerns regarding reperfusion of the brain during the TA-NRP procedure, as long as the arch vessels are clamped and the cephalad ends are vented to the atmosphere [48].

We believe that one reason for the significant divergences regarding TA-NRP is the limited global experience with this technique. In fact, in some of the countries represented by the experts in this consensus, TA-NRP has not yet been implemented. Furthermore, there is significant inconsistency in the recommendation to clamp or to ligate the aortic arch vessels (see Table 8, point 2) in order to improve the technical success of the TA-NRP, with no agreement on whether they should be also sectioned. On the contrary, when the focus is on prevention of brain perfusion during TA-NRP (Table 13), there was consensus that it is precluded by clamping or ligation of the aortic arch vessels and sectioning of the cephalic ends of the vessels, plus drainage and aspiration of blood from their caudal ends to achieve atmospheric pressure in the arteries is sufficient to maintain cessation of cerebral perfusion.

This study found broad consensus on how to assess grafts during A-NRP or TA-NRP (Table 10) and how to perform the sequence of a multiorgan donation with NRP, as well as on how to successfully retrieve the heart and lungs using TA-NRP.

There was a great consensus in the recommendations regarding the evaluation of organs in donors undergoing NRP, with routine biopsies for the evaluation of kidneys and liver considered not necessary. There was a wide consensus in the use of serial transaminases and serial lactate as useful ancillary test to assess liver function during NRP as was also detailed in the recent ELITA consensus report [17]. However, the systematic use of EVLP for the evaluation of lungs in DCDD donors remains a matter of considerable debate. Certain programs report a high proportion of lungs being assessed with EVLP [49], whereas others employ this modality only infrequently [18, 19, 50, 51]. The authors of this report considered that EVLP should be recommended for lung evaluation or reconditioning only in special cases, and not for routine cases (Table 10, point 2).

Since the use of TA-NRP is still in its early stages, different protocols exist regarding how to assess the heart and how to coordinate its maintenance with that of the other organs [25, 50, 52]. There was a clear agreement that when optimal cardiac function is achieved, TA-NRP should be discontinued as soon as possible to facilitate the recovery of the stunned heart (Table 9).

One of the major challenges in TA-NRP for the panelists were those situations where the procedure involves multiorgan recovery, which could risk the other grafts because of problems with the heart. The panelists agreed that a designated person in the team should ideally be responsible for determining that TA-NRP has been unsuccessful and shifting the team’s focus to the recovery of lungs and abdominal organs. This named person has demonstrated a great benefit in Spain, where the donor transplant coordinator, mostly an intensive care physician, has a pivotal role in organizing the different surgical teams participating in NRP procedures, particularly when TA-NRP is used [9].

The ethical concerns regarding NRP have substantially impeded the adoption of this technique worldwide [53, 54]. It was notable that more than 90% of panellists disagreed with a series of normative and empirical claims that are often presented as justifications for prohibiting NRP (Table 12). It is evident that the most critical ethical concern relates to the respect for the “dead donor rule”, with consensus among panellists that interventions are essential to ensure that the permanency standard for the determination of death is maintained during TA-NRP by preventing cerebral reperfusion.

There was a consensus that effective techniques must be used to maintain permanent cessation of cerebral perfusion and the delivery of oxygenated perfusate to the brain during NRP. Indeed, clamping or ligation of the aortic arch vessels and sectioning of the cephalic ends of the vessels, plus drainage and aspiration of blood to atmospheric pressure during TA-NRP was considered sufficient to ensure this. However, ligation of the arch vessels alone was not considered an effective technique to maintain permanent cessation of brain circulation during TA-NRP (Table 13). There is thus a critical need for research that will establish interventions that conclusively demonstrate the absence of brain perfusion after TA-NRP [55]. Two recent studies monitoring intracerebral blood pressure circle of Willis and using perfusion scintigraphy with technetium-99m hexamethylpropyleneamine oxime as a radiotracer during TA-NRP provided the first strong evidence that permanent cessation of brain perfusion could be maintained by clamping and sectioning the supra-aortic vessels [39, 46]. It remains unclear whether clamping of the supra-aortic vessels in isolation, which has been considered acceptable in some parts of the U.S, excludes any risk of postmortem cerebral perfusion [45, 46]. The US guidelines accepted both options [47].

Emerging evidence regarding the absence of cerebral perfusion during TA-NRP [39, 46], together with recent studies providing direct evidence of the temporal relationship between the loss of brain blood flow, brain activity, and systemic circulation in humans [56], helps strengthening the confidence within the scientific community—and may facilitate a more widespread adoption of NRP in the setting of DCDD.

## Data Availability

The raw data supporting the conclusions of this article will be made available by the authors, without undue reservation.
